# Digital media recommendation system design based on user behavior analysis and emotional feature extraction

**DOI:** 10.1371/journal.pone.0322768

**Published:** 2025-05-19

**Authors:** Ting Ruan, Qian Liu, Yanxizi Chang

**Affiliations:** School of art and design, Hubei University, Wuhan, Hubei, China; National Textile University, PAKISTAN

## Abstract

In the contemporary digital era, multimedia platforms, such as social media, online comment sections, and forums, have emerged as the primary arenas wherein users articulate their sentiments and viewpoints. The copious volume of textual data generated by these platforms harbors a wealth of emotional insights, which are paramount in comprehending user behaviors, fine-tuning content dissemination strategies, and elevating user satisfaction. This scholarly paper introduces an innovative framework, denominated ATLSTM-PS, for formulating content dissemination strategies within digital media platforms predicated upon a user-centric emotional perspective. Initially, it accomplishes extracting emotional content from users’ commentaries on digital media platforms, amalgamating the ATT-LSTM method with the attention mechanism, resulting in enhanced feature extraction precision compared to traditional single RNN and LSTM approaches. Subsequently, the framework extracts information at the feature layer by integrating user behavioral and emotional attributes. Following this, by amalgamating user behavioral and emotional features, ATLSTM-PS affects the synthesis of feature layer information. This meticulous amalgamation yields highly precise recommendations that cater to user demand. Empirical results obtained from publicly available and proprietary datasets substantiate that ATLSTM-PS substantially enhances the efficacy of content dissemination through the synergy of distinct attention layers. This research contributes not only a novel technical tool in sentiment analysis but also furnishes a potent methodology for multimedia platforms to refine their information dissemination strategies, thereby augmenting the user experience.

## 1 Introduction

Over the preceding decades, multimedia platforms have experienced a swift and transformative evolution, progressing from the nascent stages of static content distribution to the present-day dynamic and interactive media. These platforms have metamorphosed into an indispensable facet of individuals’ daily existence. Facilitated by technological advancements and the burgeoning Internet user base, these platforms have not only proliferated in number but have also exhibited remarkable enhancements in content diversity, richness, and interactivity. Contemporary multimedia platforms now serve as more than mere conduits for information dissemination; they profoundly influence users’ entertainment preferences, socialization patterns, and information acquisition. In modern society, they have emerged as pivotal channels for the circulation of information and cultural exchange [1].

Within this evolving landscape, artificial intelligence (AI) technologies, notably in text sentiment analysis, have forged novel avenues for comprehending user conduct and enhancing user engagement. Sentiment analysis, an essential research avenue in natural language processing (NLP), endeavors to discern and extract emotional inclinations embedded within textual data through comprehensive analysis. In recent years, deep learning methodologies, exemplified by Convolutional Neural Networks (CNN), Recurrent Neural Networks (RNN), Long Short-Term Memory Networks (LSTM), and the cutting-edge Transformers model, have gained widespread adoption in text sentiment analysis. These methodologies have significantly elevated the precision and efficiency of sentiment classification owing to their robust feature extraction capabilities and advanced learning mechanisms [2]. Furthermore, the integration of the attention mechanism has further augmented model performance. This enhancement allocates distinct degrees of importance to various text segments, enabling the model to more accurately capture emotional nuances within the text and deliver more precise sentiment analysis outcomes [3].

Text messaging is a versatile and direct conduit for users to express their emotions, offering a flexible means to communicate feelings and viewpoints. This medium facilitates sentiment analysis, which in turn aids in comprehending and addressing user needs, thereby elevating the quality of personalized services and overall user experience. The critical role of user sentiment in shaping the design of push platforms necessitates an in-depth examination [4]. The precise identification of user sentiment augments the relevance and personalization of content recommendations and substantially elevates user satisfaction and engagement levels. In light of these considerations, our research emphasizes advancing the efficacy of text sentiment analysis by utilizing natural language processing techniques and the fusion of sentiment features. This approach is subsequently applied to information dissemination systems tailored for multimedia platforms [5]. The precise contributions of this paper are delineated as follows:

- We introduce an ATT-LSTM text sentiment feature extraction approach, which combines the attention mechanism with LSTM methodology, designed explicitly for textual commentary data from digital media platforms. Our method exhibits notably superior recognition performance for positive comments compared to conventional techniques.
- We present ATLSTM-PS, an automated digital media content distribution system that integrates emotional features within the framework. This fusion of emotional attributes results in a marked enhancement in the model’s effectiveness in content matching
- In comprehensive digital media recommendation evaluations conducted on publicly available and internally developed datasets, the ATLSTM-PS framework consistently demonstrates outstanding performance. Notably, it achieves push matching rates exceeding 80% in our self-constructed dataset, thus substantiating its significant superiority over sentiment feature approaches devoid of fusion.

The structure of this article is as follows: Section 2 reviews related work, Section 3 introduces the proposed method and algorithm, Section 4 presents the experimental results and analysis, and Section 5 discusses the effectiveness and potential issues of the proposed method in practical applications.

## 2 Related works

### 2.1 Sentiment analysis based on user text information

Numerous researchers in sentiment analysis have made noteworthy contributions, garnering increasing attention for their accomplishments in machine learning. In particular, they have introduced lexicons into sentiment analysis combined with machine learning, thus enriching text-based sentiment analysis. Tureny et al. [6] analyzed word similarity by incorporating pointwise mutual information while simultaneously reconstructing the lexicon’s composition by introducing polar semantics, thereby augmenting lexical richness. Yang et al. [7] extended the lexicon’s capacity to express sentiment tendencies by introducing LDA modeling tools to further extract topic words within a pre-established sentiment lexicon, which enhanced sentiment representation. Pang et al. [8] pioneered integrating machine learning techniques into binary classification tasks within sentiment analysis. They combined these methods with traditional bag-of-words approaches to improve classification outcomes. Wikarsa et al. [9] gathered an extensive dataset from Twitter comments, categorizing emotional types and employing machine learning tools for emotion classification. Following classification, they used traditional machine learning algorithms to conduct sentiment analysis, resulting in an expanded sentiment convergence range and enhanced algorithm performance. Traditional machine learning algorithms have conventionally featured simplistic one-layer networks without introducing hidden nodes to extend network capabilities. Schwenk et al. [10] enhanced language models through deep learning, significantly elevating the accuracy of semantic recognition in lengthy, complex sentences. By introducing a novel model structure, Corrado et al. [11] bolstered semantic recognition accuracy for lengthy sentences in large datasets. This structure simplified the construction of language models, incorporating one-hot encoding for the input layer and embedding weights as word representations between the input and hidden layers. Paredes-Valverde et al. [12] proposed a model in task-oriented problem solving, leveraging the amalgamation of CNN and word2vec. This innovation resulted in a substantial enhancement in model accuracy.

Through the above research, we can see that user text sentiment analysis combines traditional machine learning and deep learning methods, relying on text feature extraction and enhancing sentiment recognition ability by introducing sentiment dictionaries, topic modeling, vector representation and other technologies. Early research was mainly based on dictionary matching and traditional classification algorithms. Still, in recent years, with the rise of deep learning, methods have gradually developed towards neural networks, semantic embedding, CNN, LDA, and other directions, improving the accuracy and robustness of sentiment analysis. The core challenges of sentiment analysis lie in semantic ambiguity, multimodal emotional expression, and long-term emotional dynamics, which remain essential directions for future research.

### 2.2 Research on recommendation algorithms

The research landscape in recommendation algorithms has evolved significantly, driven by various factors, including technological advancements, the availability of vast datasets, the diversity of user preferences, and socio-ethical considerations. The continuous march of technology has empowered recommender systems to tackle increasingly complex tasks. The wealth of extensive user behavior data provides ample training material for algorithms, expanding the horizons of research possibilities. Among the classic recommendation algorithms, collaborative filtering stands out. The user-based collaborative filtering recommendation algorithm (User-based CF) identifies user groups with similar interests to a target user, recommending items favored by individuals within these groups [13]. It is particularly suited for situations where time sensitivity is paramount or when a user’s personalized preferences are not readily discernible. However, as the user base grows, the computation of user similarity becomes increasingly intricate, often resulting in challenges in delivering rational recommendations.

Furthermore, this algorithm encounters issues related to scalability, data sparsity, and the “cold start” problem. A progression of methods has been developed to address data sparsity, beginning with the SVD algorithm based on matrix singular value decomposition [14]. Subsequently, the SVD++ algorithm was introduced, which considers domain influence [15]. Later developments introduced probability distribution functions into matrix decomposition, giving rise to PMF and BPMF algorithms [16]. Deep learning-based recommendation models have also emerged as a significant advancement. These models enhance representational capabilities by altering the complexity of neural networks. For instance, the Deep Crossing model [17], a pioneering deep learning recommendation model, employs a three-layer architecture consisting of an Embedding layer, a multi-hidden layer, and an output layer. It performs automatic deep feature cross-computation. The NeuralCF model [18], another neural network collaborative filtering model, redefines how item and user vectors interact. Additionally, models like the Wide&Deep model [19] construct a deep learning network by combining the traditional LR model and DNN model, leveraging the strengths of both for feature combination and fitting. An evolution of this approach is the DeepFM model [20], which replaces the Wide module with the FM model, enhancing the model’s feature combination capabilities.

The prevailing research landscape in text sentiment analysis and recommendation algorithms underscores the effectiveness of leveraging deep learning and machine learning techniques for text sentiment analysis. Additionally, reinforcing algorithmic features through sentiment feature polarity has shown significant potential in enhancing the accuracy of user behavior analysis. In recommendation algorithms, the amalgamation of models possesses inherent advantages. Utilizing multimodal user behavior data allows for more robust user clustering, enhancing the efficacy of data-driven recommendations. This approach optimizes the model’s final recommendation performance, ultimately benefiting users.

## 3 Methodology

### 3.1 LSTM with attention for the emotion recognition

In addressing the inquiry of emotional analysis, this paper undertakes the task of emotion classification through the LSTM methodology, grounded in textual attributes. Concurrently, it employs the attention mechanism to augment model performance, enhancing the precision of emotion recognition [21]. Within this section, we shall commence with an exposition on LSTM. LSTM) represents a variant of the RNN engineered to combat the issues of gradient vanishing and gradient explosion that afflict conventional RNNs when confronted with lengthy sequences. LSTM adeptly captures and retains protracted dependencies by introducing a series of gating mechanisms. It finds applicability across a spectrum of sequential modeling endeavors, establishing a broad presence in natural language processing. The crux of LSTM lies in incorporating three pivotal gating units: the forget gate, the input gate, and the output gate, each characterized by equations as delineated in Eqs. (1)-(3):


$$f_{t}=\sigma \left(W_{f}\cdot \left[h_{t-1},x_{t}\right]+b_{f}\right)$$ (1)

$$i_{t}=\sigma \left(W_{i}\cdot \left[h_{t-1},x_{t}\right]+b_{i}\right)$$ (2)

$$\begin{array}{cc} o_{t} & \;=\sigma \left(W_{o}\cdot \left[h_{t-1},x_{t}\right]+b_{o}\right) \end{array}$$ (3)

For a network built by LSTM, the process of information transfer inside is shown in Eqs. (4)-(6):


$${\overset{\sim }{C}}_{t}=tanh\left(W_{C}\cdot \left[h_{t-1},x_{t}\right]+b_{C}\right)$$ (4)

$$C_{t}=f_{t}\cdot C_{t-1}+i_{t}\cdot {\overset{\sim }{C}}_{t}$$ (5)

$$h_{t}=o_{t}\cdot tanh\left(C_{t}\right)$$ (6)

where $f_{t},\;i_{t},\;{\overset{\sim }{C}}_{t},\;o_{t}$ are the forgetting gate, the input gate, the candidate value of the new cell state, and the output gate’s activation value, respectively.$C_{t}$ denotes the cell state at the current time step, and $h_{t}$ denotes hidden state. w is the weight matrix, while b represents the corresponding bias. Upon the completion of text feature extraction, the imperative task entails a more profound analysis. In this pursuit, we introduce the attention mechanism to fortify the model’s capabilities. The attention mechanism manifests contextual attentiveness and the quantification of significance, effecting a targeted re-weighting operation on the input data. By assessing the likeness between the original input and output data, the attention mechanism derives weight representations, culminating in a weighted aggregation of outcomes.

In the absence of the Encoder-Decoder model, attention can be considered as a standalone entity. The computation of attention hinges upon a collection of key-value pairs. Each query undergoes a similarity assessment for a designated set of queries available for interrogation. The ultimate attention scores are determined by the outcomes of these similarity computations. The mathematical expression underpinning this calculation is elucidated in Equation (7):


$$Attention(\;query,\;Source\;)=\sum_{1}^{n}\;simality(\;query,\;key\;)*\;value\;$$ (7)

**Fig 1:**
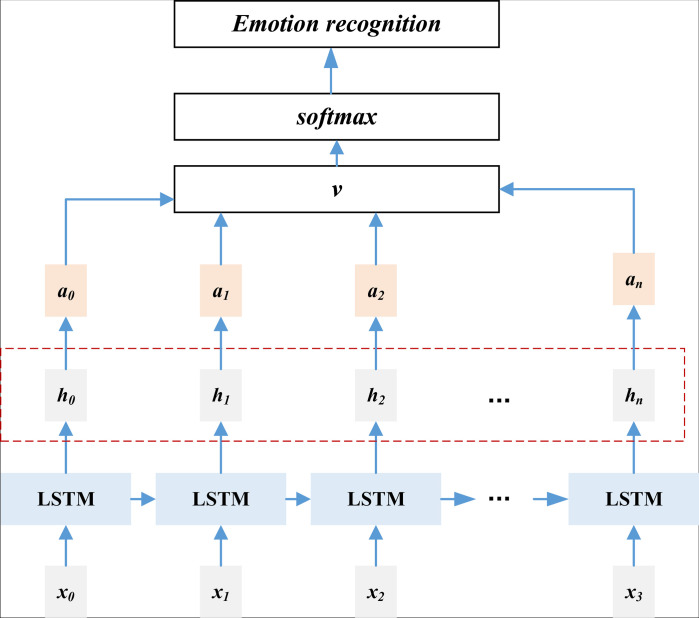
The framework for the ATT-LSTM for the text emotion recognition.

The ATT -LSTM model obtained in this paper by adding the attention mechanism is shown in Fig 1:

For the input text data $x_{0},x_{1},x_{2},\ldots ,x_{t}$, the input is passed into the LSTM network and processed by the hidden layer of the network to get the following data $h_{0},h_{1},h_{2},\ldots ,h_{t}$ After the introduction of Attention mechanism, attention is used as a mechanism to allocate the amount of attention to the data, for the data after the allocation of attention can be expressed as $\alpha_{0},\alpha_{1},\alpha_{2},\ldots ,\alpha_{t}$ After the allocation of attention, the data can be expressed as follows:, the output of the hidden layer at that moment is calculated, and the matching effect is compared to obtain the probability allocation state; the calculation formula is as follows. $\alpha_{i},j\in [0,t]$ The formula is as follows.


$$\alpha_{i}=\frac{exp\left(score\left(\overset{―}{h,}h_{i}\right)\right)}{\sum_{j}\;\;exp\left(score\left(\bar{h},h_{i}\right)\right)}$$ (8)

For each time point in the network, the ultimate goal is to compute the feature vector of the input data v:


$$v=\sum_{i=0}^{t}\;\alpha_{i}h_{i}$$ (9)

Finally, the softmax function is utilized to obtain the prediction category as y as shown in Equation (10):


$$y=softmax\left(W_{v}v+b_{v}\right)$$ (10)

Following extracting pertinent features through the LSTM framework, applying the attention mechanism yields augmented features imbued with contextual semantic nuances. Upon this enriched foundation, the ensuing sentiment classification analysis, rooted in textual content, is meticulously executed.

### 3.2 Recommendation framework incorporating attention mechanism scoring

This paper’s central issue under scrutiny revolves around the challenge of media recommendations geared explicitly toward predicting the roster of media suggestions a user is most likely to engage with—a quintessential Click-Through Rate (CTR) predicament. CTR serves as a pivotal metric in gauging the efficacy of advertisements or recommender systems, representing the probability that a user will indeed click on a particular advertisement or recommended content after its presentation. The ensemble of candidate media stems from an initial filtration process applied to the original media data during the recall phase [22].

Traditional recommendation models exhibit a modus operandi where the input layer concurrently ingests user feature vectors, historical behavioral data, and candidate media into the neural network. The objective is to extract higher-order features latent within the data and subsequently compute the probability of a user’s engagement with the candidate media. However, real-world scenarios furnish users with historical behaviors with varying informational significance. Effective recommendation processes necessitate a methodical treatment of these distinct behaviors to optimize the quality of recommendations.

**Fig 2:**
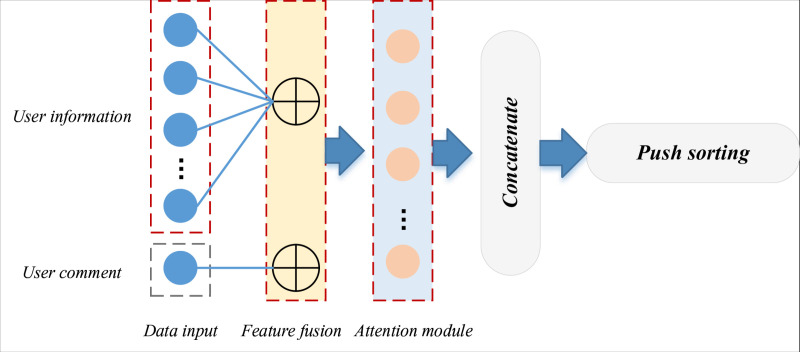
The ATLSTM-PS push sorting framework based on the emotion feature fusion.

Hence, this paper pioneers a harmonious amalgamation of the emotion recognition framework, expounded upon in the preceding subsection, and the attention mechanism. This amalgamation augments the emotional features, culminating in creating the ATLSTM-PS push framework. This novel framework is depicted in Fig 2:

The framework established extends beyond the conventional user behavior features, extracting emotional attributes from user comments. Consequently, it incorporates the attention mechanism into the feature fusion process to enhance the final recommendation predictions. In this model, the initial step involves procuring the user’s historical behavioral feature vector and the feature vector corresponding to candidate media. Subsequently, attention weights are computed for the user’s historical interactions with relevant media. Ultimately, the weighted historical user engagement history and the candidate content are input into the fully connected layer to facilitate model training.

In the context of the attention mechanism employed herein, the dot product, as delineated in Equation (11), serves as the mathematical underpinning for computing attention. Herein, u signifies the historical feature vector, and v represents the feature vector associated with the candidate media:


W(u,v)=u−v (11)

The ultimate task of media recommendation is accomplished through the computation of the pertinent ranking. The whole process can be illustrated in Algorithm 1 as follows:

**Algorithm 1.** Feature fusion process

1. Obtain user’s past clicked news/media feature vectors $U=\left\{u_{1},u_{2},\ldots ,u_{n}\right\}$

2. Obtain the feature vector *v* of the candidate news/media to be recommended.

3. Use dot-product attention to calculate relevance scores between each historical feature W(u,v)=u·v

4. Normalize the attention scores using the Softmax function:$\;\alpha_{i}=\frac{exp\left(W\left(u_{i},v\right)\right)}{\sum_{j}\;\;exp\left(W\left(u_{j},v\right)\right)}$

5. Compute the weighted sum of historical behavior features

$\overset{\sim }{u}=\sum_{i}\;\alpha_{i}u_{i}$

6. Concatenate the weighted user history vector u ~ with the candidate media vector v to create the final input for prediction:$z\;=\;\;[\overset{\sim }{u},v]$.

7. Fully connected layer classification and model training

Further considering the label imbalance in the model training, We apply weighted loss functions to compensate for the class imbalance. Given the class distribution in the dataset, we compute inverse frequency-based weights for each sentiment class and integrate them into the loss function. The adjusted loss function is defined as:


$$L=-\sum_{c}\;w_{c}\cdot y_{c}log\left({\overset{\circ }{y}}_{c}\right)$$ (12)

where $w_{c}=\frac{1}{\text{\textasciitilde{}freq(c)\textasciitilde{}}}$ represents the weight for class c, ensuring that underrepresented sentiment labels contribute more significantly to model training.

## 4 Experiment result and analysis

Once the model construction is concluded, the subsequent phase involves a comprehensive evaluation of the associated datasets. To this end, we have chosen two distinct dataset types for analysis: the MovieLens 20M Dataset (ml-20m) [23] and the MovieLens Latest Datasets (ml-latest) [24]. The ml-20m dataset, a publicly accessible repository of movie ratings, comprises feedback from 138,000 users concerning 27,000 movies. Among these ratings, there are 20,000,000 reviews and 465,000 markers. Importantly, it ensures that every user has assessed a minimum of 20 films. Conversely, the ml-latest dataset guarantees that each user has rated at least one movie.

Having established the dataset selection, this paper conducts a comparative analysis of relevant models. Specifically, it compares three fundamental approaches for emotion recognition: LSTM, CNN, and GRU. The user ratings are categorized into two groups: positive and negative reviews. Precision, recall, and F1-score metrics are employed to assess the quality of reviews. Furthermore, for satisfaction with media recommendations, we scrutinize the similarity between the top five recommendations and user-devalued media scoring data within the public dataset. The model hyperparameters setting is shown in Table 1.

**Table 1:** The model hyperparameters setting.

Hyperparameter	Value/Range	Description
Time Steps (T)	10	Defines the length of the input sequence, determining how many past interactions are considered.
Hidden Units (H)	256	Specifies the number of neurons in the LSTM layers, affecting model capacity.
Learning Rate (η)	0.001	Controls the step size in optimization; learning rate decay is applied to improve convergence.
Batch Size (B)	32	Determines the number of samples per training iteration, balancing efficiency and stability.
Dropout Rate (p)	0.2	Prevents overfitting by randomly deactivating neurons during training.

These hyperparameters were selected based on preliminary experiments and optimized using grid search and Bayesian optimization to achieve the best performance. The experiment environment is established as shown in Table 2:

**Table 2:** The experiment environment Information.

Item	Specifications
OS	Win 10
CPU	AMD Ryzen 7
GPUs	RTX3060
Language	Python 3.7
Framework	Pytorch

### 4.1 Experimental results of emotional polarity analysis

**Fig 3:**
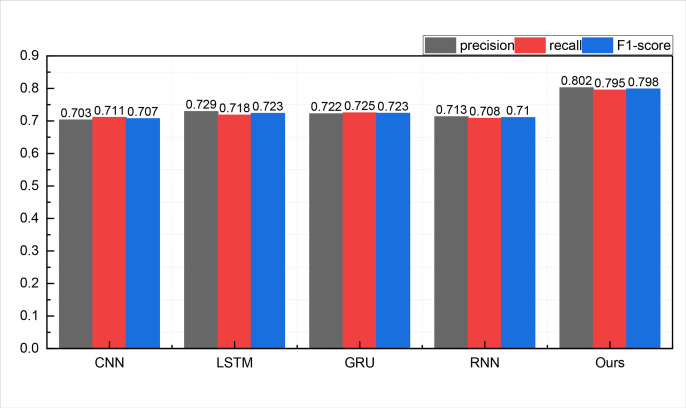
The comparison result for the emotion recognition on ml-20m dataset.

**Fig 4:**
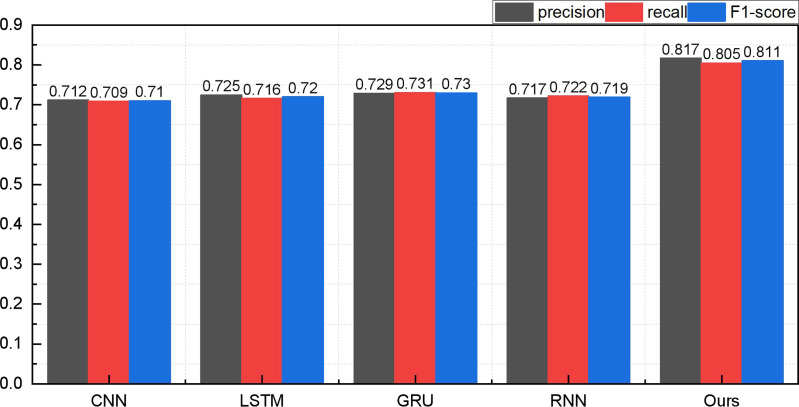
The comparison result for the emotion recognition on ml-latest dataset.

Upon the completion of model construction and the validation of recommendation success metrics, the subsequent phase encompasses model training and related assessments. Before evaluating the recommender system model, we scrutinize the efficacy of the proposed ATT-LSTM approach in emotion recognition. Given the primary focus of this paper on media recommendation, we shall exclusively present the outcomes of positive emotions. The results of positive emotion recognition in the two datasets are visually depicted in Figs 3 and 4:

In Fig 3, it is evident that the proposed method achieves a markedly superior positive emotion recognition performance compared to a standalone text emotion recognition method. It attains a recognition accuracy of 0.802, as measured by the PRECISION metric, which significantly outperforms the individual LSTM method at 0.729 and other approaches like RNN and CNN. The recognition outcomes in another dataset, namely, the ml-latest dataset, are elucidated in Fig 4:

Fig 4 reaffirms the proposed method’s superior performance, even when applied to a new dataset. Within this dataset, the fusion of word vectors is facilitated by incorporating the attention mechanism at the feature layer, resulting in a heightened precision in emotion recognition. Notably, the positive emotion precision score of 0.817 achieved under the ml-latest dataset surpasses the performance of other methods, further accentuating the method’s advantages. In stark contrast, when the attention mechanism is not integrated, the standalone LSTM method’s recognition accuracy languishes at a mere 0.725, underscoring the substantial disparity vis-à-vis the technique presented in this paper. Although the model performs well, errors occur in sarcasm detection, ambiguous sentiments, and cold-start cases. Future improvements could involve explainable AI for better sentiment interpretation and reinforcement learning for adaptive recommendations, enhancing accuracy and robustness in complex sentiment scenarios.

### 4.2 Analysis of push results incorporating emotional features

**Fig 5:**
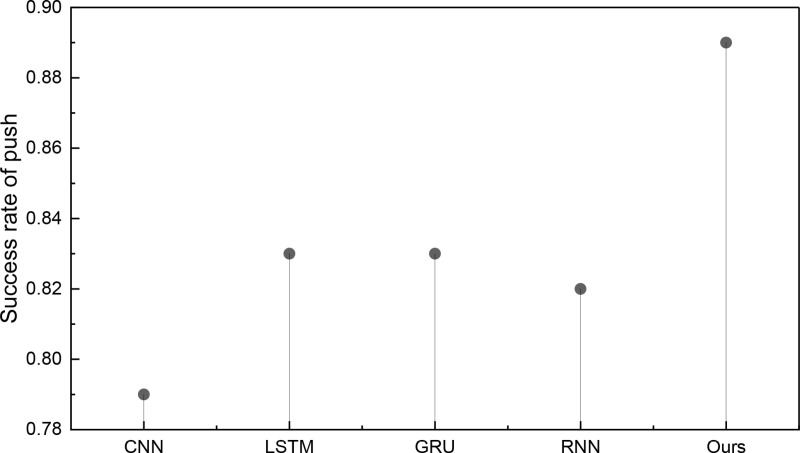
The success rate of push on ml-20m dataset.

**Fig 6:**
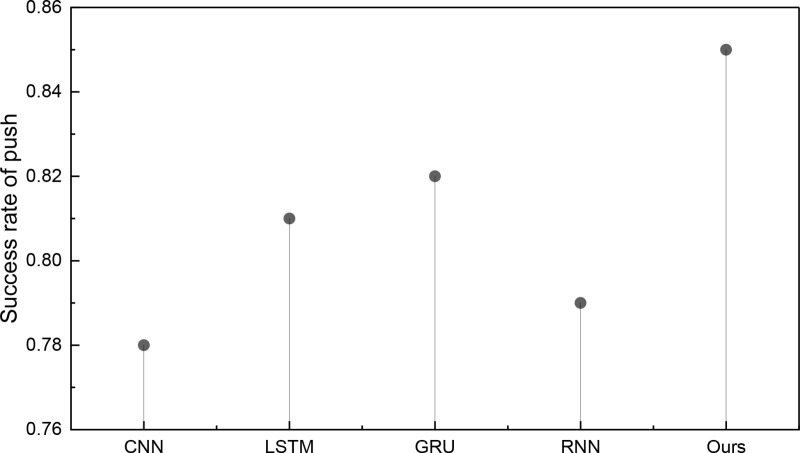
The success rate of push on ml-latest dataset.

In this applied research endeavor, we introduced the push accuracy concept and successfully devised pertinent push content strategies. Moreover, we have conducted an exhaustive analysis of the push effectiveness within the two datasets. The outcomes of this analysis are succinctly illustrated in Figs 5 and 6:

Fig 5 aptly illustrates that the ATLSTM-PS model proposed attains an impressive average success rate of approximately 90% in push matching. This noteworthy achievement underscores its capacity to consistently achieve high push success rates across various users, effectively meeting the demands of accurate multi-task push scenarios. Our push efficiency findings for another dataset are depicted in Fig 6:

Fig 6 demonstrates that, while the push success rate experiences a slight reduction, it still outperforms traditional methods. The ATLSTM-PS model, distinguished by incorporating a two-stage attention mechanism, consistently surpasses the payoffs achieved by single LSTM methods and RNN. The successful push rate under the ml-latest dataset remains above 85%, underscoring its efficacy in multi-task push endeavors. To better analyze the characteristics of push efficiency under different datasets, we conducted corresponding statistical tests to distinguish whether the efficiency under different methods is significant. The results are shown in Table 3:

**Table 3:** The significance analysis among different methods.

Method	ml-20m(mean)	ml-latest(mean)	p-value
CNN	0.703	0.712	0.066
LSTM	0.729	0.725	0.073
GRU	0.722	0.729	0.075
RNN	0.713	0.717	0.059
Ours	0.802	0.817	0.068

According to the models with different push effects in the figure, it can be seen that there is no significant difference in the results between the two datasets, indicating that this method has better applicability.

### 4.3 Practical testing of the model

Following the comprehensive testing of the model on public datasets, we assessed its performance in real-world application scenarios. First, we employed web crawlers to collect regional media data, thus assembling a self-constructed dataset. Subsequently, we executed a recommendation system that tailors its recommendations according to user ratings. Each user contributed five comments along with relevant information, and subsequent feature extraction facilitated an assessment of the algorithm’s recommendation satisfaction. This process allowed us to gauge the performance of the method.

**Fig 7:**
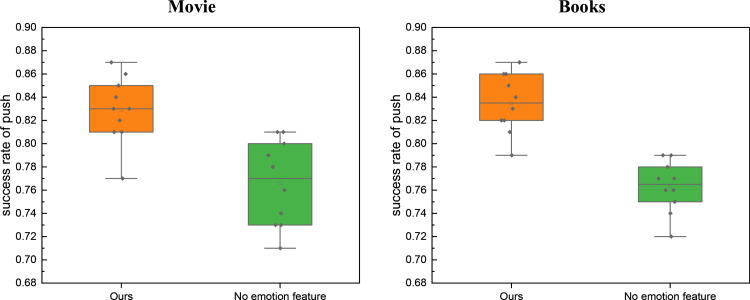
The Ablation experiment for the push success on the established dataset.

To better comprehend the impact of emotional features on the model’s performance, we analyzed user-favored movies and books for recommendation. Given the limited data capacity of our self-built database, this study focuses on a representative sample of ten users within the test set. The results of their push matching are presented in Fig 7.

Fig 7 provides compelling evidence that the proposed method, which incorporates emotional features into the model, consistently yields a high push success rate for movie and book media across various users. This underscores the significance of emotional features, particularly identifying positive sentiment in user comments, for enhancing future media recommendations.

Furthermore, the proximity of the push results between these two distinct media categories suggests that the influence of reviews is relatively uniform and exerts a similar impact on recommendations for both types of media. In the movie category, the average push matching score for the selected users stands at 0.829, notably superior to the score of 0.766 achieved without the separate inclusion of emotional features. In contrast, the recognition accuracy with and without emotional feature augmentation for the books category is 0.835 and 0.763, respectively. This demonstrates that the independent extraction of emotional features plays a more pronounced role in elevating the model’s recognition accuracy, yielding scores of 0.835 and 0.763 for the books category, respectively.

## 5 Discussion

This research delves into digital media recommendations and introduces the ATLSTM-PS push model, representing a fusion of textual sentiment feature layers. This model stands out for its remarkable advancements in textual sentiment recognition. It leverages the attention mechanism inherent in LSTM to analyze user comments’ sentiment. Moreover, it combines this sentiment feature with the user’s historical data, enabling the network to prioritize vital information within the text. This enhancement bolsters sentiment analysis accuracy and enhances the model’s sensitivity, making it markedly superior in discerning subtle sentiment nuances compared to conventional CNN, RNN, and standalone LSTM approaches. The model’s nuanced sentiment recognition capability is pivotal in elevating the success rate of user media recommendations. This, in turn, leads to a substantial augmentation in push personalization and precision. In terms of push efficiency and accuracy, the study attains a commendable push success rate exceeding 80% by adeptly interpreting users’ emotional responses and historical behavior patterns. This efficient push mechanism enriches user-profiles and is a reliable foundation for the push system’s decision-making, markedly enhancing the relevance of content recommendations and user satisfaction. Consequently, this research showcases technical innovation and carries significant implications for elevating user experience and engagement. The ATLSTM-PS model primarily relies on historical user behavior and sentiment features for personalized recommendations. However, the model employs a content-based recommendation strategy for cold-start users who lack sufficient interaction data to mitigate performance degradation. Specifically, in the absence of behavioral history, recommendations are generated based on the similarity between the semantic representations of candidate news articles and general user preference patterns derived from aggregated sentiment trends. Additionally, user demographic information (if available) can serve as an auxiliary signal to refine initial recommendations. Future work will explore integrating meta-learning or hybrid recommendation techniques, such as leveraging collaborative filtering and reinforcement learning, to enhance adaptability to cold-start scenarios.

Sentiment analysis is pivotal in the evolution of multimedia platforms, primarily due to its ability to provide deep insights into user emotions and preferences. These insights serve as the bedrock for powering personalized recommendation systems. Through precise examination of users’ emotional reactions to content, platforms can more effectively curate and recommend material that aligns with users’ interests, thereby enhancing user satisfaction and fostering greater user engagement. Sentiment analysis empowers platforms to capture the subtle fluctuations in users’ emotions and adapt content recommendation strategies accordingly, resulting in more precise targeting of users. The utilization of the ATLSTM-PS model, as introduced in this paper, not only heightens the accuracy of sentiment analysis but also augments the efficiency and precision of multimedia platforms in delivering content recommendations. By delving into the emotional inclinations expressed in user comments and integrating these emotional features with historical user data, this method furnishes platforms with richer and more comprehensive user profiles, enabling highly personalized content delivery. The successful application of this approach not only drives innovation within multimedia platforms at the technical and service levels and serves as a crucial pillar for enhancing user experience and platform competitiveness.

Furthermore, the application of intelligent sentiment analysis within multimedia platforms exerts a profoundly positive influence on user psychology. This is chiefly manifested in the elevation of personalized experiences, the enhancement of user satisfaction, and the strengthening of a sense of community belonging [25–29]. This technology empowers platforms to comprehend better and respond to users’ emotional needs, offering users more intimate and valuable content recommendations. Consequently, it bolsters users’ goodwill and loyalty toward the platform, fostering a healthy community atmosphere [30]. This, in turn, holds immense significance for the long-term development of multimedia platforms.

## 6 Conclusion

In this paper, we delve deep into the design of information push systems within multimedia platforms, introducing a novel framework called ATLSTM-PS that combines the power of the attention mechanism with the LSTM. By employing the ATT-LSTM method to precisely extract emotional information from user comments and integrating user behavioral characteristics for efficient information fusion, this study marks a significant advancement in enhancing the accuracy of user recommendations on digital media platforms. The experimental results underscore the substantial superiority of the ATLSTM-PS framework over traditional methods, such as RNN and LSTM, both on public and self-built datasets. The ATT-LSTM method consistently achieves an average recognition precision exceeding 80% in sentiment recognition. Similarly, in the context of ATLSTM-PS-based interest-driven recommendations, the push matching accuracy consistently surpasses 80% across multiple datasets. This serves as concrete evidence that this research offers a fresh perspective in sentiment analysis and personalized recommendations at the technical level and furnishes multimedia platforms with an effective means to optimize information push strategies and enhance the overall user experience.

This research contributes to news recommendation by integrating sentiment features with user behavior analysis, leveraging attention mechanisms and deep learning models to enhance recommendation accuracy. Theoretically, it extends existing work by incorporating sentiment-aware user modeling, demonstrating how emotional tendencies influence personalized recommendations. The proposed approach also contributes to feature fusion techniques by balancing sentiment and behavioral information through weighted attention mechanisms. Practically, this model provides a more refined and personalized news recommendation system, improving user satisfaction by considering emotional responses to content. It can be applied in various domains, such as customized content curation, e-commerce recommendations, and social media engagement analysis. Future implementations could optimize real-time dynamic recommendations by incorporating long-term sentiment trends and adaptive learning strategies.

This study mainly focuses on users’ emotional state at specific time points to enhance the characterization of current news interests rather than analyzing emotional trends over a long period. Although long-term emotional changes may impact recommendation systems, the core goal of this study is to optimize personalized recommendation performance by combining real-time emotional features with user behavior. In future work, we will consider introducing long-term window sentiment analysis to further explore the impact of dynamic changes in user emotions during long-term interactions on recommendation systems. Future research endeavors will be dedicated to further enhancing the ATLSTM-PS framework’s performance, particularly in comprehending intricate user emotions and behavioral intricacies. As user data grows and user needs become increasingly diverse, the information push system encounters heightened demands. This necessitates our model to exhibit greater flexibility and adaptability. Consequently, our forthcoming work will explore more advanced model architectures and learning algorithms to handle larger-scale data and conduct more nuanced user sentiment analysis. These efforts will be instrumental in ensuring that our framework remains at the forefront of addressing the evolving landscape of multimedia platforms and user requirements.
